# Special Issue “Progress in Research on Endocrine-Disrupting Chemicals”

**DOI:** 10.3390/ijms27010039

**Published:** 2025-12-19

**Authors:** Efthymia Kitraki

**Affiliations:** Laboratory of Basic Sciences, Faculty of Dentistry, School of Health Sciences, National and Kapodistrian University of Athens (NKUA), 11527 Athens, Greece; ekitraki@dent.uoa.gr

Endocrine-disruptive chemicals (EDCs) are human-made substances that mimic the action of endocrine hormones and can lead to adverse health effects in living organisms, including humans. Adversity is particularly harmful in developing organisms due to their immature detoxification systems and can lead to the life-long reprogramming of hormone-dependent functions. Epidemiological studies provide significant evidence on human exposures to different types of EDCs during different life stages. Initial risk assessment approaches have defined the safety limits of human exposure to single chemicals. However, given that organisms are exposed to various chemicals at the same time, there is a need to define relevant mixtures of EDCs that simulate real-life exposures. The defined mixtures, further examined in animal and/or ex vivo models, help to delineate the molecular mechanisms behind adversity and the exposure rates that lead to pathology. Emerging combined approaches, including epidemiological studies, enable more precise estimations of risk assessment for the protection of living organisms [1].

Current research on EDCs is characterized by multi-disciplinarity [2] and this is reflected in the articles in this Special Issue.

Contributions include two experimental studies conducted in animal models, namely, zebra fish (contribution 1) and mice (contribution 2), one study conducted ex vivo in human granulosa cells (contribution 3), a case–control study (contribution 4), and a review article on human cancer risk upon exposure to xenoestrogens (contribution 5).

At this point, we would like to express our sincere gratitude to the authors, peer reviewers, and editorial team whose contributions have made this Special Issue possible.

The study by Stradtman et al. (contribution 1) investigates the impact of exposure to the pesticide Atrazine (AΤΖ) on the neuroendocrine and neurotransmission systems of zebra fish, a well-established animal model for investigating developmental toxicity. AΤΖ exposure included two time windows, while assessments of hormonal and gene expression levels were conducted at different time points post fertilization. The authors report that embryonic ATZ exposure modified estradiol and dopamine levels both in embryos and in adult animals. Among the several genes studied, significant alterations were also detected in the expression of genes *fshb* and *gh1*, implicated in the examined neuroendocrine pathways. The results of this study show that embryonic exposure to ATZ exerts both immediate and long-term effects on the neurohormonal and neurotransmission systems of zebra fish, some of which are sex-specific.

The study by Rinotas et al. (contribution 2) examines the effects of in utero exposure to a mixture of EDCs on the mandibular growth of mice. The mixture’s components and relevant concentrations had been previously determined based on epidemiological observations in the SELMA mother–child cohort, showing that this mixture, detected in pregnant women, was associated with language delay in their neonates. The mixture has also been shown to modify brain physiology and behavior in mice exposed in utero. The results of the present study show that the same mixture also affects the growth of other structures of neural crest origin, such as the mandibular bone and condyle. This is the first study to report impaired development in mandibular components in a mammalian model upon prenatal exposure to an epidemiologically defined EDC mixture.

The study by Celar Šturm et al. (contribution 3) meticulously investigates the effects of both environmentally relevant and toxicological BPA concentrations, over different exposure times, on human granulosa cells isolated from waste follicular fluid from women undergoing in vitro fertilization. The toxicological BPA dose reduced Progesterone and Estradiol synthesis and cell viability in vitro, while all BPA doses altered the expression and protein levels of different steroidogenesis- and apoptosis-related genes in these cells. Furthermore, in compliance with the non-monotonic effects of EDCs, exposure to the lowest BPA dose modified the expression of genes related to responses to oxidative and mitochondrial stress, which merits further investigation.

The case–control study by Cao et al. (contribution 4) examines the potential association of Triclosan (TCS) and Triclocarban (TCC) exposure levels with the risk of developing type 2 diabetes mellitus in general adult Chinese populations with or without the disease. The authors provide evidence of such an association, as well as of an association with the levels of glucose-metabolism indicator markers. Furthermore, the study examines the potential mediating effect of SOCS3 methylation on the detected associations. The results of this study provide valuable evidence for associations between the parameters investigated and suggest further research in the field of estrogen-mimicking synthetic antimicrobials, used worldwide in personal care products.

The comprehensive review article by Gachowska et al. (contribution 5) explores human cancer risk upon exposure to various xenoestrogens. Xenoestrogens (XEs) are exogenous molecules that mimic estrogens’ actions. However, their signaling and decay are not appropriate, leading to disfunction in the endocrine system. Aberrations range from reproductive and metabolic complications to cancer development. Due to the wide array of XE-related pathologies, there is need for this knowledge to be updated. The authors provide data for the most common XEs, including information for their estrogenic/anti-estrogenic activity and the molecular pathways through which their actions may lead to common hormone-dependent cancers. Furthermore, the article refers to the implications of XEs in disfunction in critical organs, such as the uterus, lungs, and kidneys.

Overall, the articles in this Special Issue reflect the range of multifaceted contemporary research approaches in the field of EDCs. The main objective in current research is to define all adverse outcomes resulting from real-life exposures to mixtures of these compounds. The accurate scaling up of risk assessment from model organisms to humans is important in preventing exposures leading to pathology. Furthermore, recent progress in personalized mixture toxicology, which estimates risk based on an individual’s mixture exposure [3], is expected to provide more precise information to guide health protection.
